# Identification of ovule transcripts from the Apospory-Specific Genomic Region (ASGR)-carrier chromosome

**DOI:** 10.1186/1471-2164-12-206

**Published:** 2011-04-26

**Authors:** Yajuan Zeng, Joann Conner, Peggy Ozias-Akins

**Affiliations:** 1 Department of Horticulture, The University of Georgia Tifton Campus, Tifton, GA, 31973, USA

## Abstract

**Background:**

Apomixis, asexual seed production in plants, holds great potential for agriculture as a means to fix hybrid vigor. Apospory is a form of apomixis where the embryo develops from an unreduced egg that is derived from a somatic nucellar cell, the aposporous initial, via mitosis. Understanding the molecular mechanism regulating aposporous initial specification will be a critical step toward elucidation of apomixis and also provide insight into developmental regulation and downstream signaling that results in apomixis. To discover candidate transcripts for regulating aposporous initial specification in *P. squamulatum*, we compared two transcriptomes derived from microdissected ovules at the stage of aposporous initial formation between the apomictic donor parent, *P. squamulatum *(accession PS26), and an apomictic derived backcross 8 (BC_8_) line containing only the Apospory-Specific Genomic Region (ASGR)-carrier chromosome from *P. squamulatum*. Toward this end, two transcriptomes derived from ovules of an apomictic donor parent and its apomictic backcross derivative at the stage of apospory initiation, were sequenced using 454-FLX technology.

**Results:**

Using 454-FLX technology, we generated 332,567 reads with an average read length of 147 base pairs (bp) for the PS26 ovule transcriptome library and 363,637 reads with an average read length of 142 bp for the BC_8 _ovule transcriptome library. A total of 33,977 contigs from the PS26 ovule transcriptome library and 26,576 contigs from the BC_8 _ovule transcriptome library were assembled using the Multifunctional Inertial Reference Assembly program. Using stringent *in silico *parameters, 61 transcripts were predicted to map to the ASGR-carrier chromosome, of which 49 transcripts were verified as ASGR-carrier chromosome specific. One of the alien expressed genes could be assigned as tightly linked to the ASGR by screening of apomictic and sexual F_1_s. Only one transcript, which did not map to the ASGR, showed expression primarily in reproductive tissue.

**Conclusions:**

Our results suggest that a strategy of comparative sequencing of transcriptomes between donor parent and backcross lines containing an alien chromosome of interest can be an efficient method of identifying transcripts derived from an alien chromosome in a chromosome addition line.

## Background

Apomixis, asexual reproduction through seed, is widespread among flowering plant families, but low in its frequency of occurrence [1]. Different from sexual reproduction, apomictically derived embryos develop autonomously from unreduced ovular cells instead of through fertilization of a reduced egg by a sperm. Therefore, the progeny of an apomictic plant are genetically identical to the maternal plant [2,3]. This trait can be used as an advanced breeding tool in agriculture since it would enable fixation of hybrid vigor and seed propagation of desirable genotypes [4-7]. No major agriculturally important crop possesses this trait [8-10]. Introgression of apomixis into crops through crossing has been impeded by factors such as polyploidy and incompatibility [9]. Therefore, discovery of genetic mechanisms underlying apomixis will be crucial for manipulation of apomixis for introduction into target crops.

Apomixis has been classified into two types and three developmental pathways: gametophytic apomixis, including apospory and diplospory, and sporophytic apomixis, which is also known as adventitious embryony [2]. In sporophytic apomixis, an embryo forms directly from an ovular cell and coexists with the zygotic embryo. For gametophytic apomixis, the embryo develops from an unreduced egg in an embryo sac derived through mitosis of either a somatic nucellar cell (apospory) or the megaspore mother cell (diplospory). In apospory, meiosis either does not complete or its products degenerate while aposporous initials (AIs) develop from one or more somatic nucellar cells. Both genotypes chosen for the present study are aposporous with the trait conferred by genetic elements from *Pennisetum squamulatum*. Aposporous *P. squamulatum *has four-nucleate embryo sacs that lack antipodals [10]. Apospory in this species is inherited as a dominant Mendelian trait [11] and is associated with an approximately 50 Mb, heterochromatic and hemizygous chromosomal region designated the Apospory-Specific Genomic Region (ASGR), [12,13].

Many transcriptional approaches to discover the regulatory mechanisms and downstream effects associated with apomixis in many species have been undertaken. In *Brachiaria*, differential display applied to apomictic and sexual ovaries at anthesis yielded two apomixis-specific fragments [14] while a study on earlier sporogenesis and gametogenesis stages identified eleven differentially expressed fragments [15]. In *Paspalum notatum*, three expressed sequence tags (ESTs), all highly similar in sequence, showed differential expression in flowers between apomictic and sexual F_1 _individuals after apospory initiation [16]. An additional 65 genes were identified as differentially expressed between sexual and aposporous plants [17]. cDNA-AFLP analysis in *Paspalum simplex *yielded transcripts linked to the apomixis-controlling locus (ACL). Many of these linked fragments showed stop codons and frameshift mutations, suggesting that they are pseudogenes [18]. cDNA-AFLP was also applied to identify apomixis candidate genes in *Poa pratensis *where 179 transcript-derived fragments from spikelets showed qualitative and quantitative expression differences between apomictic and sexual genotypes [19]. The full-length sequences of two genes of interest, *PpSERK *(SOMATIC EMBRYOGENESIS RECEPTOR-LIKE KINASE) and *APOSTART *were obtained and their temporal and spatial expression patterns were assessed by reverse transcription polymerase chain reaction (RT-PCR) and in situ hybridization, respectively. While neither one of these two candidate genes showed apomixis- or sexual-specific expression, quantitative differences in expression between apomictic and sexual genotypes were observed [20].

One apomixis-specific gene was identified from a *Panicum maximum *ovule cDNA library and shown to be expressed in both aposporous initials and embryos at four days after anthesis [21,22]. Additional genes have been identified in *Panicum *through microarray and quantitative RT-PCR analysis [23]. In *Pennisetum ciliare*, differential display and suppression subtractive hybridization were used to identify gene expression differences in ovaries of sexual and apomictic accessions [24,25]. SuperSAGE, a high-throughput differential display approach, has been used to discover several hundred transcripts with heterochronic shifts in expression between apomictic and sexual ovules at multiple stages of development [26,27].

Formation of aposporous initials is the first and most critical event for occurrence of apospory. Because the initiation of sexual and apomictic pathways likely is activated by different signals [28], understanding the molecular mechanism underlying apospory initiation can provide insight into developmental regulation and downstream signaling that results in apomixis. In order to discover candidates for regulating aposporous initial specification in *P. squamulatum*, we compared two transcriptomes derived from microdissected ovules at the stage of aposporous initial (AI) formation between the apomictic donor parent, *P. squamulatum*, and its apomictic derivative backcross 8 (BC_8_) containing a single *P. squamulatum *chromosome. Initially, a *P. glaucum *x *P. squamulatum *F_1 _was crossed with a *P. glaucum *x *P. purpureum *F_1 _and hybrid apomictic individuals with good male fertility were selected [29]. Subsequent backcrosses with tetraploid *P. glaucum *[30] yielded a BC_8 _line that was shown by FISH to contain only one chromosome from *P. squamulatum*. This single chromosome common to both apomictic BC_8 _and *P. squamulatum *was the ASGR-carrier chromosome based on the transmission of the trait of apomixis and linked molecular markers [31]. We hypothesize that candidate genes regulating aposporous initial specification and localized to the ASGR will function in both PS26 and BC_8 _at the same developmental stage and would be identical in sequence as they are related by descent.

The development and commercialization of new massively parallel sequencing platforms have made transcriptome sequencing faster and more affordable. One platform, developed by 454 Life Sciences Corporation, the 454 GS-FLX sequencer, is capable of producing 100 Mb of sequence data with an average read length of 250 bp per bead in a 7-h run [32]. Successful applications of these high-throughput sequencing technologies to transcriptome analysis have been reported [33-37]. Here we present expressed sequence tags (ESTs) generated by Roche 454 high-throughput sequencing technology from dissected ovule tissues staged for aposporous initial formation from two apomictic lines chosen for their common features of apospory and single shared chromosome. Alien chromosome (ASGR-carrier chromosome) expressed transcripts were identified and tested for ASGR linkage and tissue expression.

## Results

### Aposporous ovule-enriched cDNA samples for sequencing

**Figure 1:**
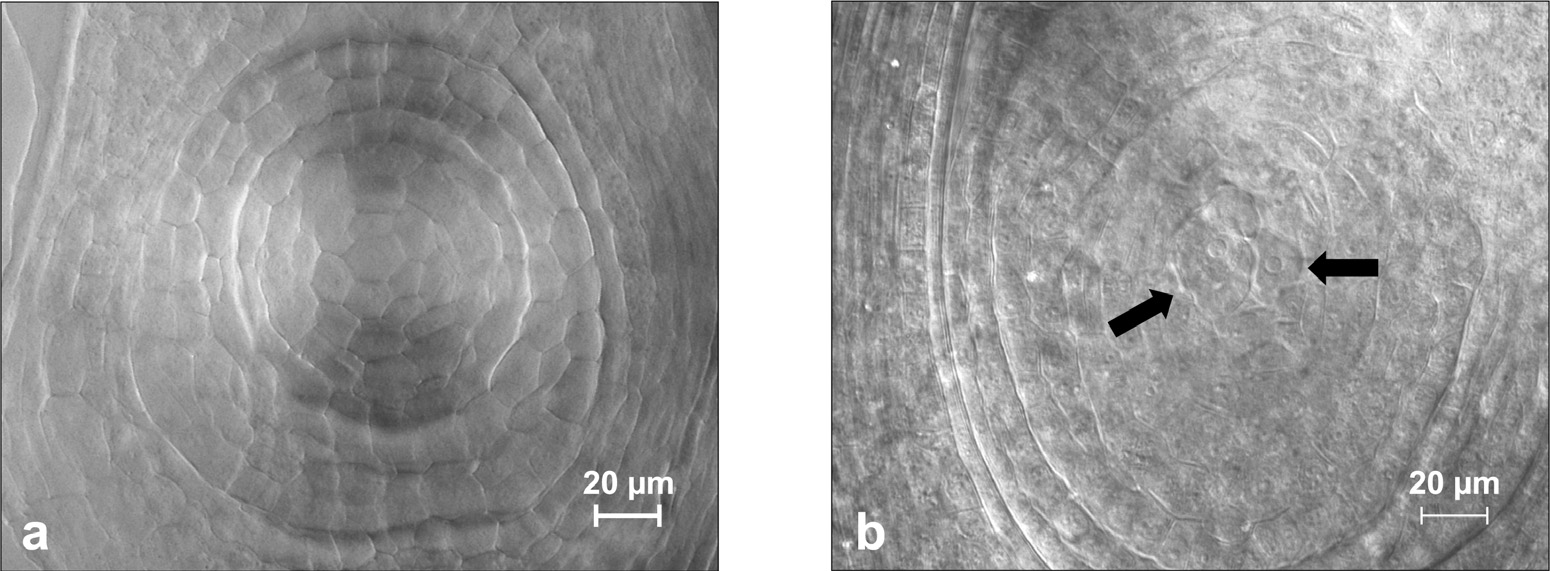
**Microdissection and ovary clearing**. a: cleared ovary showing no aposporous initials and prior to megasporogenesis. b: cleared ovary showing two aposporous initials, indicated by solid arrows.

**Figure 2:**
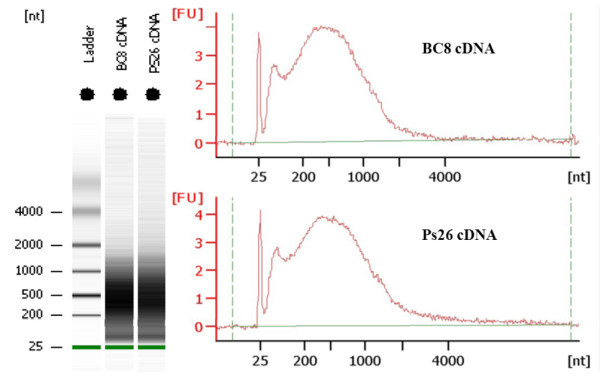
**Agilent Bioanalyzer 2100 analysis result of the ds-cDNA samples**.

Ovules from PS26 and BC_8 _around the stage of aposporous initial formation were manually dissected from pistils (Figure 1). Three biological replicates of 40 ovules each were collected for both PS26 and BC_8_. The yield of total RNA from each replicate was approximately 20 ng from which 15 ng was used for one-round of T7 RNA polymerase-based RNA amplification. The average yield from one round of amplification was 90 μg. For each library, equal amounts of amplified RNA from each replicate were combined and 15 μg amplified RNA was used for ds-cDNA synthesis. The majority of the ds-cDNA synthesized from amplified RNA was distributed in a size range from 200 bp to 1000 bp (Figure 2).

### Assembly of sequences from PS26 and BC_8 _aposporous ovules

Two aposporous ovule transcriptomes, one from PS26 and the other from BC_8_, were sequenced using the high-throughput 454-FLX sequencer. The PS26 transcriptome library contained 332,567 reads with an average read length of 147 base pairs (bp) and the BC_8 _transcriptome library contained 363,637 reads with an average read length of 142 bp. Assembly by the Multifunctional Inertial Reference Assembly (MIRA) program [38] resulted in 33,977 contigs from the PS26 ovule transcriptome library and 26,576 contigs from the BC_8 _ovule transcriptome library (Additional file 1: PS26_MIRA.fasta, Additional file 2: BC8_MIRA.fasta). The number of reads per contig ranged from 1 to 759 in PS26 assemblies and 1 to 1661 in BC_8 _assemblies with the majority having less than 30 reads per assembly in both cases. The numbers of singletons in PS26 and BC_8 _libraries were 176 and 78, respectively.

### Blast2GO

**Figure 3:**
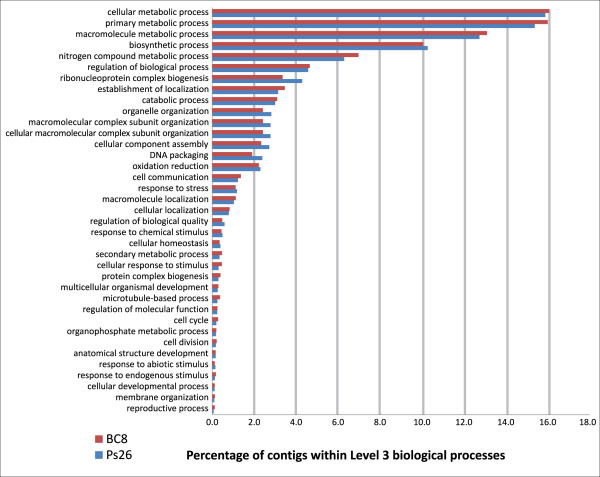
Blast2GO Level 3 biological processes for PS26 and BC_8._

Contigs from both transcriptome libraries were analyzed for biological functions using Blast2GO [39]. For both libraries, the use of T7 amplified RNA biased the sequencing data toward the 3' UTR region as shown by the BlastX results of the Blast2GO analysis. 5,730 PS26 contigs (~17%) and 4,833 BC_8 _contigs (~18%) had hits against the nr database of NCBI with an E-value cut-off of e^-06^. For both libraries, 90% of the top BlastX hits were, in order, to *Sorghum bicolor*, *Zea mays *or *Oryza sativa *proteins. Blast2GO was able to fully annotate 4,400 PS26 contigs and 3,692 BC_8 _contigs (Figure 3).

To obtain additional functional data from the shorter reads, a study was initiated to test whether the most significant BlastN EST_other database hit (E-value cut off of e^-20^) could be used as a surrogate longer sequencing read for the PS26/BC_8 _transcripts. Approximately 55% (14,518) of the BC_8 _contigs had an EST_OTHERS hit ≤e^-20^. Blast2GO analysis was used for the BC_8__EST_OTHERS best matches and compared with Blast2GO mapping results for the 3692 annotated BC_8 _contigs. The majority (84%) of the BC_8 _contigs had Blast2GO mapping data identical to the corresponding BC_8__EST_OTHERS mapping data while only 5% of the BC_8 _contigs had >50% non-matching mapping data. Given the large percentage of identical and/or highly matching mapping data, a library of PS26_EST_OTHERS was also established using the same parameters as BC_8__EST_OTHERS. Approximately 53% (18,028) of the PS26 contigs had an EST_OTHERS hit ≤e^-20^. Blast2GO was able to fully annotate 12,462 PS26_EST_OTHERS contigs and 10,107 BC_8__EST_OTHERS contigs.

A Fisher's Exact Test (using GOSSIP; [40]) was done to identify significant differences of expression data between the PS26 and BC_8 _libraries and the PS26_EST_OTHERS and BC_8__EST_OTHERS libraries. At a false discovery rate (FDR) ≤0.01, 28 GO terms were identified as different between the PS26 and BC_8 _libraries (Table 1). However, when the PS26_EST_OTHERS and BC_8__EST_OTHERS libraries were compared at FDR <0.05 (at an FDR ≤0.01 no significant results were returned), only 7 GO terms (ribosome, translation, ribosome biogenesis, ribonucleoprotein complex biogenesis, ribonucleoprotein complex, structural constituent of ribosome, cellular component biogenesis) were identified as differentially expressed between the two libraries (Table 1).

**Table 1:** GO terms within the biological process category significantly over- or under-represented between the libraries.

GO TERM ID	description	Adjusted p-value for Ps26_contigs (FDR ≤0.01)	Over/Under representation Ps26_contigs	Adjusted p-value for Ps26_EST_OTHERS contigs (FDR ≤0.05)	Over/Under representation Ps26_EST_OTHERS contigs
GO:0005840	ribosome	1.90E-05	over	1.10E-04	over
GO:0006412	translation	5.81E-06	over	1.47E-04	over
GO:0042254	ribosome biogenesis	6.05E-06	over	1.61E-04	over
GO:0022613	ribonucleoprotein complex biogenesis	7.31E-06	over	1.61E-04	over
GO:0030529	ribonucleoprotein complex	2.47E-05	over	1.73E-04	over
GO:0003735	structural constituent of ribosome	6.02E-06	over	2.05E-04	over
GO:0044085	cellular component biogenesis	9.23E-06	over	2.34E-04	over
GO:0043228	non-membrane-bounded organelle	1.07E-05	over	n.s.	
GO:0043232	intracellular non-membrane-bounded organelle	1.07E-05	over	n.s.	
GO:0005198	structural molecule activity	5.57E-05	over	n.s.	
GO:0034645	cellular macromolecule biosynthetic process	1.52E-04	over	n.s.	
GO:0032559	adenyl ribonucleotide binding	1.24E-05	under	n.s.	
GO:0005524	ATP binding	1.46E-05	under	n.s.	
GO:0032553	ribonucleotide binding	1.60E-05	under	n.s.	
GO:0032555	purine ribonucleotide binding	1.60E-05	under	n.s.	
GO:0000166	nucleotide binding	5.36E-05	under	n.s.	
GO:0001882	nucleoside binding	5.56E-05	under	n.s.	
GO:0017076	purine nucleotide binding	5.77E-05	under	n.s.	
GO:0001883	purine nucleoside binding	5.91E-05	under	n.s.	
GO:0030554	adenyl nucleotide binding	5.91E-05	under	n.s.	
GO:0003824	catalytic activity	9.79E-05	under	n.s.	
GO:0016740	transferase activity	1.38E-04	under	n.s.	
GO:0006793	phosphorus metabolic process	1.67E-04	under	n.s.	
GO:0006796	phosphate metabolic process	1.67E-04	under	n.s.	
GO:0006073	cellular glucan metabolic process	1.75E-04	under	n.s.	
GO:0044042	glucan metabolic process	1.75E-04	under	n.s.	
GO:0016773	phosphotransferase activity, alcohol group as acceptor	2.50E-04	under	n.s.	
GO:0016310	phosphorylation	3.01E-04	under	n.s.	

### *In Silico *identification of putative alien expressed transcripts

When MIRA-assembled contigs from the two libraries were analyzed by BlastN with PS26 sequences as queries and BC_8 _sequences as the database, a total of 118 comparisons were obtained with 100% sequence identity across an overlapping region ≥100 bp corresponding to 115 unique contigs from the PS26 database and 116 unique contigs from the BC_8 _database. The 118 PS26/BC_8 _contigs were further analyzed by aligning the corresponding PS26 and BC_8 _contigs with each other, resulting in 61 inter-genotype contigs with no mismatches that were aligned. The average overlapping regions of the 61 inter-genotype contigs was 241 bp (ranging from 181 bp to 419 bp) with an average number of 28 sequence reads. The remaining PS26/BC_8 _contigs, while initially identified by BlastN as having 100% identity over a region >100 bp, did not continue to share sequence similarity outside this region and therefore did not align over the whole contig.

### Mapping and predicted function of putative ASGR-carrier chromosome transcripts

**Figure 4:**
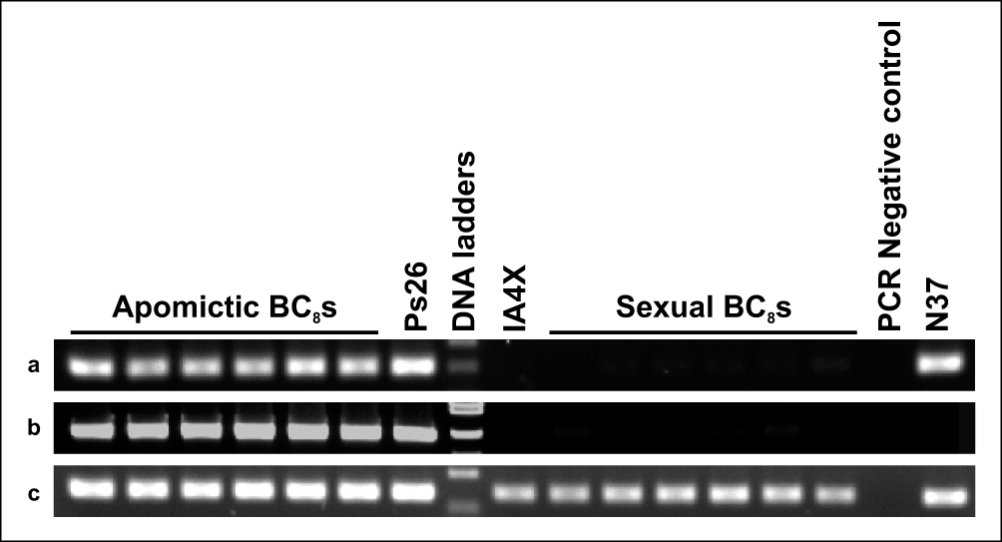
**Examples for mapping of transcripts to the ASGR-carrier chromosome**. a: amplification from PS26, N37 and apomictic BC_8_but not from IA4X or sexual BC_8_(PS26_c583: p1510/p1511). b: amplification from PS26 and apomictic BC_8_but not from IA4X, N37 or sexual BC_8_(PS26_c9369: p1514/p1515). c: amplification from PS26, IA4X, N37 and both apomictic and sexual BC_8_(no specificity; PS26_c4364: p1504/p1505). Specificity for PS26_c4364 subsequently was achieved by using a different primer pair (Table 2).

Up to four primer pairs per contig were used to test for linkage of the 61 contigs to the ASGR-carrier chromosome. Sequence characterized amplified region (SCAR) primer pairs were designed based on the PS26 contig sequence (Additional file 3, Table S1). After screening by PCR against PS26, IA4X (4 × *P. glaucum*), N37 (*P. purpureum*) and a small number of progeny from apomictic BC_8 _segregating for mode of reproduction, 45 contigs showed specific amplification from PS26 and apomictic BC_8 _but no amplification from IA4X or sexual BC_8 _individuals (Figure 4, Table 2) establishing linkage of 45 contigs to the ASGR-carrier chromosome. Single-strand conformation polymorphism analysis (SSCP) and a CAPS screen using two to four restriction enzymes was applied to the 14 primer pairs which amplified products in both PS26 and IA4X DNA. Four additional contigs could be linked to the ASGR-carrier chromosome using SSCP analysis (Table 2). The CAPS screen identified a *Hae*III polymorphism for PS26_c2552, a transcript also mapped by SSCP.

**Table 2:** Summary of mapping results

PS26 contig name	Primers	size	PS26	IA4X	N37	Transcripts mapped to the ASGR-carrier chromosome	Transcripts mapped as tightly linked to the ASGR locus
**PS26_c9369**	**1514/1515**	**274**	**+**	**-**	**-**	**Yes**	**Yes**
PS26_c10331	1476/1477	210	+	-	-	**Yes**	np
PS26_c13922	1486/1487	200	+	-	-	**Yes**	np
PS26_c5080	1506/1507 1744/1745^CAPS^	204 800	+ +	- +	- N/A	**Yes** **Yes**	np No
PS26_c2339	1528/1529	213	+	-	-	**Yes**	np
PS26_c2785	1534/1535	226	+	-	-	**Yes**	np
PS26_c194	1604/1605	283	+	-	-	**Yes**	np
PS26_c2838	1642/1643	103	+	-	-	**Yes**	np
PS26_c3609	1646/1647	150	+	-	-	**Yes**	np
PS26_c5210	1652/1653	157	+	-	-	**Yes**	np
PS26_c6744	1658/1659	202	+	-	-	**Yes**	np
PS26_c5851	1654/1655	179	+	-	-	**Yes**	np
PS26_c1406	1583/1681	250	+	-	-	**Yes**	np
PS26_c28392	1704/1705	181	+	-	-	**Yes**	np
PS26_c4364	1505/1716	150	+	-	-	**Yes**	np
	1504/1505	140	+	+	+	np	np
PS26_c11544	1478/1479	165	+	-	+	**Yes**	np
PS26_c13157	1480/1481	161	+	-	+	**Yes**	np
PS26_c13655	1482/1483	214	+	-	+	**Yes**	np
PS26_c1372	1484/1485	215	+	-	+	**Yes**	np
PS26_c2448	1492/1493	189	+	-	+	**Yes**	np
PS26_c30691	1498/1499	206	+	-	+	**Yes**	np
PS26_c3546	1500/1501	245	+	-	+	**Yes**	np
PS26_c583	1510/1511	212	+	-	+	**Yes**	np
PS26_c8165	1512/1513	150	+	-	+	**Yes**	np
PS26_c1279	1530/1531	228	+	-	+	**Yes**	np
PS26_c7587	1532/1533	172	+	-	+	**Yes**	np
PS26_c17388	1538/1539	163	+	-	+	**Yes**	np
PS26_c3455	1540/1541	102	+	-	+	**Yes**	np
PS26_c1312	1542/1543	143	+	-	+	**Yes**	np
PS26_c338	1548/1549	120	+	-	+	**Yes**	np
PS26_ c33813	1565/1566 1724/1725^CAPS^	140 900	+ +	- +	+ N/A	**Yes** **Yes**	np No
PS26_c1422	1567/1568	120	+	-	+	**Yes**	np
PS26_c6131	1571/1572	179	+	-	+	**Yes**	np
PS26_c2388	1575/1576	128	+	-	+	**Yes**	np
PS26_c32589	1581/1582	216	+	-	+	**Yes**	np
PS26_c10535	1630/1631	148	+	-	+	**Yes**	np
PS26_c2807	1640/1641	164	+	-	+	**Yes**	np
PS26_c9776	1664/1665	170	+	-	+	**Yes**	np
PS26_c6373	1656/1657	178	+	-	+	**Yes**	np
PS26_c1878	1690/1691	157	+	-	+	**Yes**	np
PS26_c19109	1692/1693	163	+	-	+	**Yes**	np
PS26_c22381	1696/1697	246	+	-	+	**Yes**	np
PS26_c4150	1650/1715	450	+	-	+	**Yes**	np
PS26_c704	1708/1709	155	+	-	+	**Yes**	np
PS26_c3993	1502/1713	800	+	-	+	**Yes**	np
PS26_c30198	1496/1497^SSCP^	210	+	+	+	**Yes**	np
PS26_c1472	1573/1574^SSCP^	185	+	+	+	**Yes**	np
PS26_c2552	1670/1671^SSCP/CAPS^	243	+	+	+	**Yes**	No
PS26_c14318	1666/1667^SSCP^	175	+	+	+	**Yes**	np
PS26_c6192	1720/1721	140	+	+	+	np	np
PS26_c20942	1488/1489	125	+	+	+	np	np
PS26_c24301	1490/1491	120	+	+	+	np	np
PS26_c25664	1494/1495	193	+	+	+	np	np
PS26_c5781	1508/1509	156	+	+	+	np	np
PS26_c2405	1577/1578	180	+	+	+	np	np
PS26_c15085	1579/1580	120	+	+	+	np	np
PS26_c1580	1628/1629	237	+	+	+	np	np
PS26_c18163	1632/1633	169	+	+	+	np	np
PS26_c3656	1648/1649	152	+	+	+	np	np
PS26_c21597	1668/1669	150	+	+	+	np	np
PS26_c8378	1662/1663	199	pf	pf	pf	N/A	N/A

The one contig mapped to the ASGR is shown in bold. +: positive amplification; -: no amplification; pf: primer failure; np: no polymorphism available for mapping; N/A: not assayed. Primer sequences and annealing temperatures can be found in Additional file 3 - Table S1.

**Figure 5:**
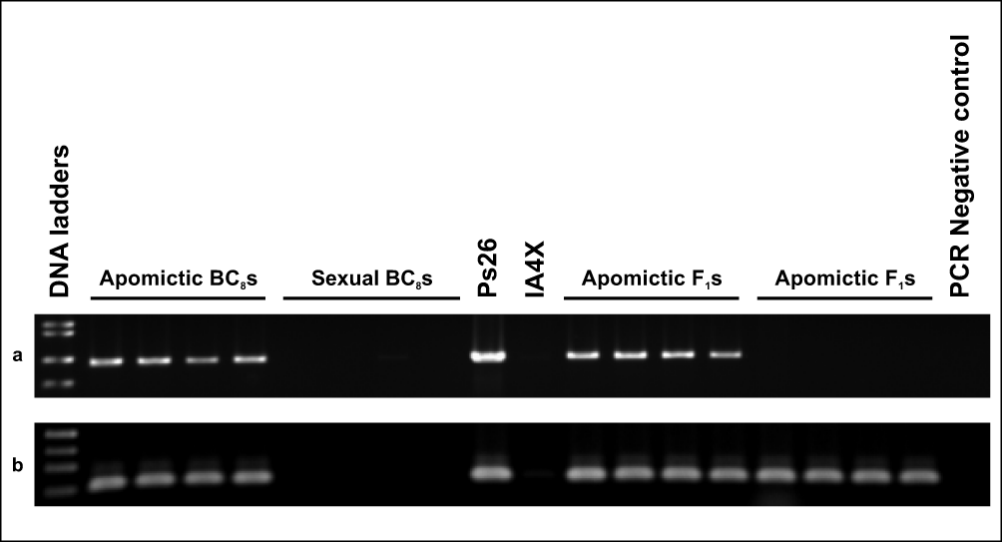
**Examples for mapping of transcripts to the ASGR**. a: amplification of apomictic F1s but not sexual F1s (PS26_c9369: p1514/p1515). b: amplification of both apomictic F1s and sexual F1s (PS26_c5080: p1506/p1507).

The markers from the 49 ASGR-carrier chromosome-linked contigs were initially screened on a limited number of apomictic (4) and sexual (4) F_1_s for mapping to the ASGR. This resulted in one contig, PS26_c9369, showing tight linkage to the ASGR as the primers amplified DNAs from only apomictic F_1_s but not sexual F_1_s (Figure 5, Table 2). The remaining primer sets did not show amplification specificity in the F_1 _population; both apomictic and sexual progeny amplified.

A larger F_1 _population of 22 individuals (10 apomictic and 12 sexual) was used to map the PS26_c9369 and PS26_c2552 transcripts. PS26_c2552 was mapped based on the *Hae*III polymorphism found in the CAPS screen between PS26 and IA4X and also seen in the F_1 _population. PS26_c2552 is unlinked to the ASGR as the CAPS polymorphism segregated 1:1 in the population but with 7 sexual and 5 apomictic individuals containing the marker. In comparison, the PS26_c9369 primers remained specific to the 10 apomictic plants and did not amplify the 12 sexual plants.

BlastX searches against NCBI databases were carried out for the 49 PS26/BC_8 _ASGR-carrier chromosome linked contigs and best protein hits for 18 contigs are summarized in Table 3. Because the sequences are 3' biased, a BlastN analysis against the expressed sequence tag (EST_OTHERS) database at NCBI with the remaining 31 PS26/BC_8 _contigs was done to find potential orthologs from other species. At an E-value cutoff of e^-20^, 18 contigs had EST hits (Table 3). A BlastX was performed using these EST sequences to determine if tentative protein functions could be obtained, and the best hits are listed in Table 3. The remaining 13 (27%) contigs did not have hits by either BlastX or BlastN; therefore, they were considered orphan genes.

**Table 3:** Potential function of transcripts mapping to the ASGR-carrier chromosome based on BlastX or BlastN.

Ps26 Contig	BC_8 _Contig	Overlap length (bp)	BlastX	BlastN (E-Value) to EST_OTHERS	BlastX of EST hit in BlastN column
Ps26_ c10331	BC_8__ c7991	241	no hit	RCRST0_005870 Foxtail millet EC612643.1 GI:149362118 (3e-55)	no hit

Ps26_ c11544	BC_8__ c10325	228	no hit	no hit	

Ps26_ c13157	BC_8__ c5112	227	no hit	no hit	

Ps26_ c13655	BC_8__ c24571	192	no hit	pPAP_06_E02 Apomictic pistil BM084376.1 GI:27532285 (8e-24)	putative 26S proteasome non-ATPase regulatory subunit 3 ACG34075.1 GI:195624490

Ps26_ c1372	BC_8__ c12789	326	no hit	CCGC4364.g1 CCGC *Panicum virgatum *early floral buds + reproductive tissue FL750787.1 GI:198007657 (e-174)	NADH-ubiquinone oxidoreductase 51 kDa subunit NP_001148767.1 GI:226532265

Ps26_ c13922	BC_8__ c12833	212	no hit	no hit	

Ps26_ c2448	BC_8__ c12858	225	no hit	pPAP_10_F04 Apomictic pistil FL813942.1 GI:198086024 (2e-57)	ankyrin protein kinase-like NP_001152470.1 GI:226495939

Ps26_ c30691	BC_8__ c10294	206	no hit	no hit	

Ps26_ c3546	BC_8__ c8622	295	no hit	no hit	

Ps26_ c5080	BC_8__ c12542	212	hypothetical protein OsJ_24918 EEE67490.1 GI:222637358		

Ps26_ c583	BC_8__ c6141	223	no hit	6X_JF-rd_A11 pAPO *Cenchrus ciliaris *EB652936.1 GI:164107582 (6e-127)	SRC2 protein kinase C -phospholipids ACG40316.1 GI:195641696]

Ps26_ c8165	BC_8__ c5964	185	no hit	84Z_JF_G03 pAPO *Cenchrus ciliaris *EB661430.1 GI:164123871 (7e-70)	TPA: AT-hook motif nuclear localized protein 2 FAA00302.1 GI:119657406

Ps26_ c9369	BC_8__ c3452	190	hypothetical protein OsJ_30933 EAZ15525.1 GI:125574241		

Ps26_ c2339	BC_8__ c7917	264	no hit	CCGG12847.g1 CCGG *Panicum virgatum *late flowering buds FL812358.1 GI:198084376 (e-23)	ESP4 (ENHANCED SILENCING PHENOTYPE 4) NP_195760.1 GI:15240970

Ps26_ c1279	BC_8__ c8634	243	ENT domain containing protein ACG36577.1 GI:195629872		

Ps26_ c7587	BC_8__ c11918	202	ATPNG1 (*Arabidopsis Thaliana *PEPTIDE-N-GLYCANASE 1) NP_199768.1 GI:15240508		

Ps26_ c2785	BC_8__ c8847	273	ubiquitin-conjugating enzyme E2 N NP_001148361.1 GI:226491078		

Ps26_ c194	BC_8__ c2920	304	no hit	no hit	

Ps26_ c17388	BC_8__ c6454	208	histone 4 BAG68513.1 GI:195972757		

Ps26_ c3455	BC_8__ c8607	193	no hit	26X_JF_C01 pAPO *Cenchrus ciliaris *EB655151.1 GI:164198597 (e-102)	putative condensing XP_002529162.1 GI:255576542

Ps26_ c1312	BC_8__ c3757	313	no hit	25X_JF_D10 pAPO *Cenchrus ciliaris *EB656417.1 GI:164027660 (2e-47)	protein phosphatase 2A regulatory subunit A AAM94368.1 GI:22296816

Ps26_ c338	BC_8__ c3527	419	universal stress protein (USP) family protein NP_001159067.1 GI:259490110		

Ps26_ c33813	BC_8__ c2708	229	putative MADS-domain transcription factor CAA70485.1 GI:3851333		

Ps26_ c1422	BC_8__ c3852	245	no hit	no hit	

Ps26_ c6131	BC_8__ c8955	224	no hit	CCHY9952.g1 CCHY *Panicum virgatum *callus FL987585.1 GI:198319427 (e-49)	putative calcium-dependent protein kinase ACG46220.1 GI:195653505

Ps26_ c2388	BC_8__ c2949	201	no hit	6W6III_JF_H03 pAPO *Cenchrus ciliaris *EB662068.1 GI:164227478 (3e-48)	polygalacturonase inhibitor 1 precursor ACG36448.1 GI:195629614

Ps26_ c32589	BC_8__ c3672	229	no hit	71Z_JF_B09 pAPO *Cenchrus ciliaris *EB654350.1 GI:163993222 (5e-93)	putative microtubule-associated protein CAD23144.1 GI:37776903

Ps26_ c10535	BC_8__ c22186	182	no hit	no hit	

Ps26_ c2807	BC_8__ c12602	241	no hit	5X_JF_A06 pAPO *Cenchrus ciliaris *EB652848.1 GI:164180053 (6e-116)	Phosphoglucomutase/phosphomannomutase C terminal ABN08987.1 GI:124361015

Ps26_ c2838	BC_8__ c3538	183	no hit	no hit	

Ps26_ c3609	BC_8__ c10814	245	no hit	no hit	

Ps26_ c5210	BC_8__ c5192	273	hypothetical protein OsJ_25077 EEE67565.1 GI:222637433		

Ps26_ c6744	BC_8__ c292	257	ADP-ribosylation factor BAB90396.1 GI:20161472		

Ps26_ c9776	BC_8__ c4965	258	no hit	MK_7_78 *Pennisetum glaucum *seedlings CD726437.1 GI:32277284 (2e-46)	hypothetical protein SORBIDRAFT_07g010440 XP_002444160.1 GI:242078783

Ps26_ c5851	BC_8__ c5854	192	no hit	no hit	

Ps26_ c6373	BC_8__ c6664	235	no hit	1475276 CERES-197 *Zea mays *FL451677.1 GI:211043870 (2e-41)	hypothetical protein LOC100276553 NP_001143786.1 GI:226505008

Ps26_ c30198	BC_8__ c9466	220	centromere/microtubule binding protein cbf5, putative XP_002523427.1 GI:255564866		

Ps26_ c3993	BC_8__ c16185	246	fk506-binding protein, putative XP_002534360.1 GI:255587693		

Ps26_ c4364	BC_8__ c15332	181	no hit	CCHZ9541.g1 CCHZ *Panicum virgatum *GD021384.1 GI:198352214 (5e-31)	helix-loop-helix-like protein AAO72577.1 GI:29367409

Ps26_ c1472	BC_8_8_ c3819	330	small zinc finger-like protein AAD40002.1 GI:5107180		

Ps26_ c1406	BC_8__ c4551	221	putative anther ethylene-upregulated protein ER1 BAC79907.1 GI:33146619		

Ps26_ c1878	BC_8__ c7425	242	no hit	CCGI4193.g1 CCGI *Panicum virgatum *FL856163.1 GI:198128193(3e-69)	hypothetical protein SORBIDRAFT_02g036200 XP_002460850.1 GI:242045958

Ps26_ c19109	BC_8__ c9186	205	no hit	no hit	
Ps26_ c22381	BC_8__ c547	185	no hit	2X6III_JF-rd_A11 pAPO *Cenchrus ciliaris *EB652659.1 GI:164076750 (7e-58)	APx2 - Cytosolic Ascorbate Peroxidase ACG41151.1 GI:195643366

Ps26_ c28392	BC_8__ c12100	230	no hit	no hits	

Ps26_ c4150	BC_8__ c3261	276	rRNA-processing protein EBP2, putative XP_002526440.1 GI:255570978		

Ps26_ c704	BC_8__ c1322	368	26S protease regulatory subunit, Putative XP_002526219.1 GI:255570523		

Ps26_ c2552	BC_8__ c1808	384	40S ribosomal protein S6 ACG31980.1 GI:195620300		

Ps26_ c14318	BC_8__ c14583	366	triose phosphate/phosphate translocator ACG33816.1 GI:195623972		

Potential functions of the inter-genotype contigs sharing 100% identity between PS26 and BC_8 _ovule transcripts which could be mapped to the ASGR-carrier chromosome based on the best hit of the contigs to protein (BlastX) or nucleotide (BlastN) sequences in NCBI databases.

In order to generate contiguous sequence that might enhance the potential for mapping of contigs in the F_1 _population and to extract a longer cDNA sequence for PS26_c9369, a cDNA library containing ~300,000 phage plaques was constructed from apomictic BC_8 _mature ovary and anther RNA since all 49 ASGR-carrier chromosome transcripts showed expression in these tissues by RT-PCR. Screening of the cDNA library with 27 ASGR-carrier chromosome transcript probes yielded hybridization signals for 24 probes. PCR screening with the ASGR-carrier chromosome-specific primers identified 16 ASGR-carrier chromosome clones and one clone for PS26_c9369. Additional sequence for these clones was generated.

The PS26_c9369 clone contained a 646 bp insert. BlastX analysis identified similarity to a hypothetical protein SORBIDRAFT_10g020450 (XP_002438482.1; e-value 6e^-18^) and *Oryza sativa *hypothetical protein OsJ_30933 [EAZ15525.1; e-value 4e^-16^] over an ~155 bp region. In both sorghum and rice, the area of similarity overlapped a pfam03004: Transposase_24 domain for those proteins. The remaining PS26_c9369 clone sequence was unique. Nine primer sets were designed from nine PS26 contigs to span introns based on predicted splicing of best hits to sorghum. Five primer sets gave strong amplification of PS26 genomic DNA. These amplicons were cloned and sequenced to identify SNPs within the PS26 genomic alleles. CAPS markers could be designed for PS26_c1580 (*Hpy*CH4IV) and PS26_c33813 (*Hpy*CH4IV). Mapping of 4 apomictic and 4 sexual F_1_s did not show tight linkage of these contigs to the ASGR.

### Expression profiles of ASGR-linked expressed transcripts by RT-PCR

**Figure 6:**
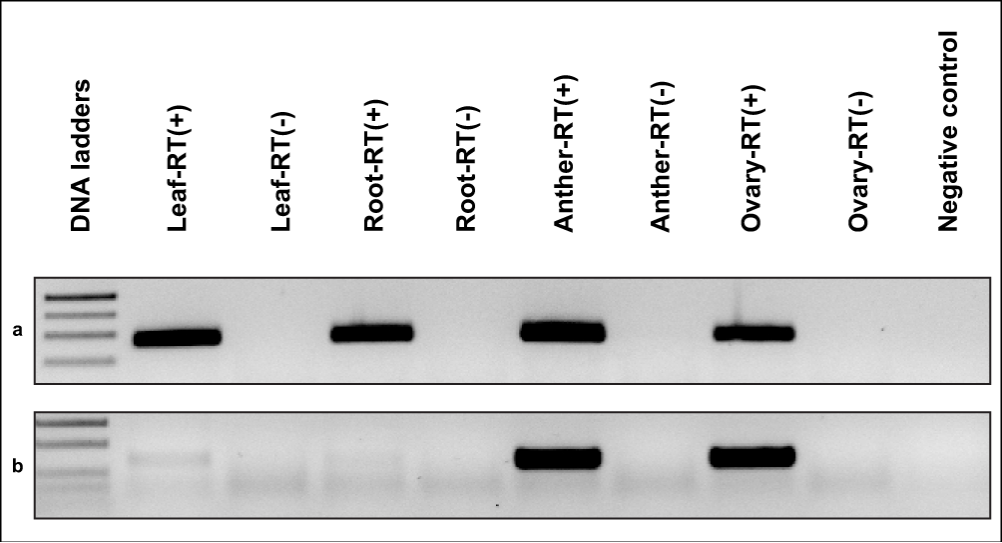
**Examples of expression patterns for ASGR-carrier chromosome linked sequences**. a: most genes showed expression in all four organs tested (Ps26_c194: p1604/p1605). b: one gene was expressed in only ovary and anther (PS26_ c33813: p1565/p1566). RT(+): RT with reverse transcriptase; RT(-): RT without reverse transcriptase as DNA contamination control.

RT-PCR with RNA extracted from apomictic BC_8 _leaf, root, anther, and ovary tissues was completed for the 49 candidate genes mapped to the ASGR-carrier chromosome. Forty-seven were expressed in all four organ types examined (Figure 6a). However, one putative MADS-domain containing transcription factor, corresponding to contig PS26_c33813, showed amplification only in anther and ovary tissues (Figure 6b) and contig PS26_c10535, a putative Lon protease, showed expression in all organs except anther.

## Discussion

Transcriptional profiling has been extensively used for gene discovery in plants because the absence of introns greatly enhances the information content of the data set and eases data interpretation [41-43]. Combined with 454 high-throughput sequencing technology, transcriptome sequencing has become an approach to understand molecular events at the gene expression level on a genome-wide scale. Many successful applications of 454 sequencing technology in transcriptome sequencing and single nucleotide polymorphism (SNP) discovery have been reported [44-49] and supported our use of this technology for ovule transcriptome sequencing.

In contrast to studies aimed at identifying genes involved in apomictic reproduction through the identification of differences between apomictic and sexual genotypes, our study compared two apomictic lines for identical transcripts. We previously reported that the ASGR is sufficient to induce apomixis in sexual pearl millet [11,12]; therefore, the trait of apomixis in BC_8 _is conferred by the ASGR-carrier chromosome from PS26 [31]. In the present study, we have attempted to identify candidate genes regulating the first step of apomixis, aposporous initial development, by transcriptome analysis of ovules from both PS26 and BC_8_. The ovules were collected at the stage of aposporous initial development, which ranged from no apparent apospory initials (~70%) to distinct aposporous initials observed (~30%). By pooling ovules over this range of development our objective was to minimize the chance of missing genes involved in the pathway of apomixis initiation since we would predict transcription prior to, and perhaps beyond, apospory initial formation.

The two ovule transcriptomes generated had an average read length of ~150 bp, shorter than the average read length of 200-300 bases for the 454 GS FLX sequencer. The shorter than expected reads could have been due to a combination of factors in preparing the samples for sequencing such as the T7-based antisense RNA amplification method, the conversion of antisense RNA to cDNA, or during the shearing process of the cDNA to prepare the sequencing library. Another possible factor is the species itself. It has been shown that the average read length can vary among different organisms due to differences in AT/GC content [32].

Even with short reads and using stringent comparison conditions to decrease the number of false positive joins between highly similar but not identical transcripts from the two species, 61 putative ASGR-carrier chromosome candidate expressed genes were identified *in silico*, of which 49 have confirmed linkage to the ASGR-carrier chromosome. The 3' bias of the T7 amplified transcripts helped in the design of primers to discriminate between *P. squamulatum *and the BC_8 _pearl millet genome containing one *P. squamulatum *chromosome. Our sequencing strategy helped remove, at least to a chromosomal level, the difficulties associated with candidate gene identification by comparative gene expression analysis in apomictic and sexual systems which lack, due to the apomictic process, an ability to generate isogenic lines that vary only in their mode of reproduction. Primer specificity for 48 transcripts was not seen when we attempted to map SCARs to the ASGR using a F_1 _population containing many *P. squamulatum *chromosomes. The additional sequence generated by the phage cDNA clones allowed mapping of two more transcripts in the F_1 _population. Greater sequence length would be advantageous for mapping of the ASGR-carrier chromosome transcripts to the ASGR locus.

The use of the gene ontology software Blast2Go allowed comparison of both the PS26 and BC_8 _libraries and the PS26_EST_OTHERS and BC_8__EST_OTHERS libraries created by using the most significant EST_OTHERS BlastN result as a surrogate for our sequences. The PS26 and BC_8 _transcriptomes were almost identical on a level 3 biological process comparison. While many biological GO terms showed expression level differences when comparing the PS26 and BC_8 _libraries, all but seven became non-significant when the PS26_EST_OTHERS and BC_8__EST_OTHERS libraries were compared. Six of the transcriptional differences noted belong to genes involved in either ribosomal or translational functions. This difference may be caused by ploidy level difference of PS26 (an octoploid) and BC_8 _(a tetraploid). MIRA assembly will separate alleles of genes into different contigs. More PS26 allelic transcripts for genes involved in either ribosomal or translational functions may be expressed in PS26 than in BC_8 _thus leading to a higher transcript difference between the libraries.

Expression analysis of the ASGR-carrier chromosome linked genes in BC_8 _tissue was used to identify transcripts specific to reproductive tissue. All but two ASGR-carrier chromosome transcripts showed constitutive expression in both vegetative and reproductive tissues. The one reproduction-specific transcript (the MADS box gene) did not map to the ASGR. The transcript which could be mapped to the ASGR shows similarity to "hypothetical" proteins in both sorghum and rice containing a Transposase_24 domain. Previous sequencing of BAC clones linked to the ASGR have shown a large number of both Type I and Type II transposons at the locus [50,13]; therefore, it is not surprising that we identified an ASGR-linked transposon transcript in our study.

## Conclusions

Our data show that the combination of selecting specific reproductive tissues and sequencing with 454 high-throughput sequencing technology is a promising approach for identification of genes involved in different developmental events and that a need for longer transcript contigs will be a requirement to allow for easier mapping of these transcripts. Given the rapid advancements in next-generation sequencing technologies that enable very deep sequence coverage and paired-end reads, it is likely that the fine tissue dissection requiring RNA amplification of starting materials now could be eliminated to favor longer transcript assemblies.

## Methods

### Plant materials

*Pennisetum squamulatum *(PS26; PI 319196, 2n = 56) and backcross line 8 (BC_8_)-line 58were used for ovule collection. Compared with the BC_7 _line which was used in previous studies [12], the BC_8_-line 58 contains only one alien chromosome from PS26, the ASGR-carrier chromosome [31]. *P. glaucum *(IA4X), *P. purpureum *(N37), 4 apomictic and 4 sexual plants from BC_8_-line 58(BC_8 _is facultative thus it produces ~ 18% sexually derived offspring were used for assigning the candidate transcript fragments to the ASGR-carrier chromosome. Twenty-two individuals from a segregating F_1 _population between *P. squamulatum *and *P. glaucum *were used for mapping the transcript fragments to the ASGR.

### RNA isolation

Young florets were dissected from small inflorescence sections whose anthers were at stages between premeiosis and prophase, as determined by acetocarmine staining of anther squashes. One group of florets was stored in RNA*Later*^® ^solution (Ambion, Austin, TX, USA) at 4°C while the other group was processed for ovary clearing by methyl salicylate [51] to screen for the ovary developmental stage. Ovules from thirty cleared florets were examined for each group. If the cleared sample showed AIs in less than 30% of the ovaries and the remaining ovaries were at an earlier developmental stage, then florets stored in RNA*Later*^® ^solution from the same section of inflorescence were used for ovule dissection. About 40 ovules per sample were collected and total RNA was extracted from the ovules with RNAqueous^®^-Micro Kit (Ambion). RNA integrity and quantity were analyzed with an Agilent 2100 Bioanalyser (Santa Clara, CA) at the Interdisciplinary Center for Biotechnology Research (ICBR) of the University of Florida.

### RNA amplification and ds-cDNA synthesis for Roche 454 sequencing

With total RNA as starting material, mRNA was amplified by T7-based in vitro transcription following the manual of TargetAmp™2-Round aRNA Amplification Kit 2.0 (Epicentre, Madison, WI). Size range and quantity of the amplified mRNA were measured by both gel electrophoresis and Agilent 2100 Bioanalyser analysis. For each sample, an equal amount of amplified mRNA from the three biological replicates was pooled for ds-cDNA synthesis following the protocol developed by the Schnable lab [52]. Size-range and quantity of ds-cDNA were also analyzed by both gel electrophoresis and using the Agilent 2100 Bioanalyser before submitting the samples for sequencing.

### 454 sequencing and processing

About 6 μg of ds-cDNA from both PS26 and BC_8 _was submitted to the Genome Sequencing Center at Washington University for 454-FLX sequencing. Samples of cDNA were subjected to mechanical shearing (nebulization), size selected, and blunt-end fragments were ligated to short adaptors, which provided primer target sites for both amplification and sequencing. Sequencing files (Accession #SRA030528) were submitted to the Sequence Read Archive at NCBI http://trace.ncbi.nlm.nih.gov/Traces/sra/sra.cgi?view=studies. The Multifunctional Inertial Reference Assembly (MIRA) program [38] was used to process and assemble the sequences from each library. Adaptor sequences and low quality sequence reads were removed prior to assembly. The assembly was run as a *de novo*, 454 EST project with accurate assembly and polyA/T clipping. Each library of contig assemblies from PS26 and BC_8 _was converted to a database and analyzed with the BlastN program provided by the RCC (Research Computing Center) at the University of Georgia http://rcc.uga.edu. The PS26 library contigs were chosen as queries and the BC_8 _library was chosen as the database. The BlastN analysis was performed with an E-value cutoff of ≤ e^-100^. The BlastN output was parsed using an internal script such that only contigs with 100% identity over at least 100 bp were selected for further analysis.

### BLAST analysis of the selected contigs

BlastX was used to analyze sequences mapping to the ASGR-carrier chromosome by searching against the NCBI (National Center for Biotechnology Information, http://www.ncbi.nlm.nih.gov/) databases. A BlastN analysis was conducted on contigs without significant BlastX hits (e-value ≤ e^-06^) to search for similar ESTs from other species. The most significant EST hit with an e-value of at least ≤e^-20 ^was used for BlastX query to search for putative encoding proteins.

### Mapping of identical PS26/BC_8 _contigs to the alien chromosome and/or ASGR

Fasta files containing sequences from contigs with 100% identity over at least 100 bp from both PS26 and BC_8 _libraries were generated. Alignment of each PS26/BC_8 _contig pair yielded sixty-one assemblies of PS26/BC_8 _contigs used as candidates for mapping to the ASGR-carrier chromosome. The 61 PS26/BC_8 _contigs from were used as queries with BlastN against both the PS26 and BC_8 _MIRA-assembled databases at an E-value cutoff of ≤e^-25^. The BlastN results were parsed and used to help estimate the 'uniqueness' of the contig within the transcriptome. Primers were designed based on the overlapping region of PS26 and BC_8 _contigs, and in some cases included further 3' sequences for primer design if the contig was unique in both databases. When multiple contigs from each database showed high similarity to each other, primers were designed based on the region with the best polymorphisms to distinguish one from another. Primers were first tested for amplification with PS26, IA4X, N37 and 4 apomictic and 4 sexual plants from a segregating population of BC_8_. Primer pairs which did not amplify either IA4X or sexual BC_8 _individuals were used for further screening with apomictic and sexual F_1_s to test for linkage to the ASGR.

For SSCP analysis a Bio-Rad Protean II system (Bio-Rad Laboratories, Hercules, CA) was used to separate fragments in a 1 mm thick 12% non-denaturing PAGE gel with 10% glycerol. PCR product (2 μl) was mixed with 10 μl LIS loading dye (10% sucrose, 0.01% bromophenol blue, and 0.01% xylene cyanol FF), denatured at 98°C for 10 min and cooled to RT for at least 10 min. Sample (10 μl) was loaded and the gel was run in at 200 V for 20-22 hours at 25°C. Silver staining was used to detect the SSCP fragments.

### Expression patterns of transcripts mapped to the alien chromosome

Total RNA was extracted from a panel of BC_8 _tissues including vegetative (leaf, root), and reproductive tissues at anthesis but before pollination (anther and ovary) with QIAGEN RNeasy^® ^Plant Mini kit (QIAGEN, Valencia, CA) following the manufacturer's protocol. First-strand cDNA was synthesized following the manufacturer's protocol of First-strand cDNA Synthesis kit (Invitrogen, Carlsbad, CA). RT-PCR reactions were performed using primer pairs which mapped to the ASGR-carrier chromosome in a total volume of 20 μl containing 1 μl of first-strand cDNA, 1 μM of each primer, 1X PCR buffer, 1.5 mM MgCl_2_, 0.2 mM dNTPs, and 1 unit of JumpStart™ *Taq *DNA polymerase (Sigma, St. Louis, MO). Amplification of contaminating genomic DNA was tested by the inclusion of controls that omitted the reverse transcriptase enzyme from the cDNA synthesis reaction, e.g. no RT controls. The PCR reaction was denatured at 94°C for 5 min followed by 35 cycles of 94°C denaturation for 30 seconds, annealing for 30 seconds at respective temperatures, and 72°C extension for 1 min. RT-PCR products were separated on a 1.5% agarose gel and stained with ethidium bromide. Gel images were captured with the Molecular Imager Gel Doc XR System (Bio-Rad Laboratories).

### cDNA library construction

Ovaries and anthers collected from apomictic BC_8 _around anthesis but prior to fertilization were frozen in liquid nitrogen. Total RNA was extracted with the RNeasy^® ^Plant Mini kit (QIAGEN) and then poly A^+ ^RNA was purified from total RNA with Oligotex^® ^mRNA Mini kit (QIAGEN) following the manufacturer's protocols. Yield of mRNA was quantified with a Nanodrop spectrophotometer (Thermo Fisher Scientific Inc., Wilmington, DE). mRNA was used for double-stranded cDNA synthesis with ZAP-cDNA^® ^Synthesis Kit following the manufacturer's protocol (Stratagene, La Jolla, CA). Ligations, packaging, titering of the packaging reactions, and plaque lifts were conducted following the manufacturer's protocol of ZAP-cDNA^® ^Gigapack^® ^III Gold Cloning Kit (Stratagene).

### cDNA library screening for target genes

The apomictic BC_8 _ovary and anther-enriched cDNA library was screened with α-^32^P labeled probes with transcripts mapping to the ASGR-carrier chromosome. The PCR fragments amplified from apomictic BC_8 _genomic DNA with the primers used for assigning a fragment to the ASGR-carrier chromosome were diluted and labeled with α-^32^P by PCR in a total volume of 20 μl. The labeling reaction contained ~0.1 ng primary PCR fragment, 1.25 unit Jumpstart *Taq *DNA polymerase (Sigma), 0.25 μM of each primer, 0.5 mM dATP/dTTP/dGTP mixture, 5 μl of α-^32^P-labeled dCTP (3000 Ci/mmol) and 1 × PCR buffer (10 mM Tris-HCl, 50 mM KCl, 1.5 mM MgCl_2_). Probes were purified by passing through homemade Sephadex G-50 (Sigma) columns, which were assembled with Ultrafree^®^-MC Centrifugal Filter Units (Millipore, Bedford, MA). Pre-hybridization of the membranes in hybridization buffer (0.5 M sodium phosphate, 7% SDS, 1 mM EDTA, pH 8.0) containing 0.1 mg ml^-1 ^salmon sperm DNA, which was denatured in boiling water for 10 minutes and cooled on ice before adding to the hybridization solution, was conducted at 65°C for 4 h before addition of the labeled, denatured probe. Hybridization was conducted at 65°C overnight followed by three washes at the same temperature for 30 min each with the following buffers: 1) 1 × SSC, 0.1% SDS; 2) 0.5 × SSC, 0.1% SDS; 3) 0.1 × SSC, 0.1% SDS. After the final wash, membranes were wrapped with plastic film and exposed to x-ray film overnight at -80°C prior to manually developing with Kodak^® ^GBX Developer and Fixer (Thermo Fisher Scientific Inc). Autoradiographs were aligned with the respective plates to recover hybridizing plaques with sterile glass pipettes. Recovered plaques were released in tubes containing 1.0 ml SM phage buffer (according to the formula in the manual of ZAP-cDNA^® ^Gigapack^® ^III Gold Cloning Kit) and 20 μl chloroform (Sigma). After overnight elution at 4°C, 1 μl SM buffer of each recovered sample was used for PCR to verify positive signals. Since the primary screening was carried out with a high density of plaque clones, the recovered positive plaques were purified after secondary and tertiary screens at much lower densities. Single plaques showing positive hybridization signals were recovered in 500 μl SM buffer with 10 μl chloroform (Sigma) at 4°C.

### Sequencing and mapping of candidate cDNA clones to the ASGR

In vivo excision of single plaque clones was conducted using ExAssist^® ^helper phage with SOLR^® ^strain following the protocol in the manual of ZAP-cDNA^® ^Gigapack^® ^III Gold Cloning Kit (Stratagene). Single colonies containing the pBluescript double-stranded phagemid with the cloned cDNA insert were isolated and cultured in liquid Luria-Bertani (LB) medium containing 100 μg mL^-1 ^ampicillin at 37°C overnight. An aliquot of each culture was further grown in freeze broth containing 100 μg mL^-1 ^ampicillin at 37°C overnight and then stored at -80°C before sending out for sequencing. Sequencing was conducted with M13 primers (Georgia Genomics Facility, Athens, GA). Vector and bad quality sequences were trimmed from the original sequences with VectorNTI Advanced 10 (Invitrogen) and primers were designed with VectorNTI using the high quality cDNA sequences. Primers were then tested with apomictic and sexual F_1_s for linkage to the ASGR as described above.

### Blast2GO

Annotation for each library was performed using Blast2GO software, http://www.blast2go.org/start_blast2go[39]. BlastX (database: GenBank nr/E-value cutoff: e^-06^), GO term mapping (default values) and Annotation (database: b2g-2009 with default values) were used. Annotations were validated and augmented using ANNEX. Libraries were compared using the Fisher's exact test with FDR value of ≤0.01 or ≤0.05.

## Authors' contributions

YZ and JC performed the sequence analysis. YZ collected and prepared cDNA samples for 454 sequencing. YZ and JC mapped transcripts and did expression analysis. JC performed the Blast2GO analysis. PO-A provided guidance for the study. All authors have read and approved the manuscript.

## Supplementary Material

Additional file 1 **PS26_MIRA.fasta**. A fasta file containing the MIRA assembled contigs of the PS26 ovule transcriptome. Click here for file

Additional file 2 **BC8_MIRA.fasta**. A fasta file containing the MIRA assembled contigs of the BC_8_ovule transcriptome. Click here for file

Additional file 3 **Table S1 - Primers designed for mapping transcripts to the ASGR-carrier chromosome**. Microsoft word file: ASGR-Carrier Chromosome transcript primers.doc contains a table with primer sequences used for experiments to map ovule transcripts to the ASGR-carrier chromosome and the ASGR locus with annealing temperatures. Click here for file
