# Supplementary material for: Identification of ovule transcripts from the Apospory-Specific Genomic Region (ASGR)-carrier chromosome
Source: BMC Genomics. 2011 Apr 26;12:206. doi: 10.1186/1471-2164-12-206 (PMC3111391; doi:10.1186/1471-2164-12-206)
Supplement: Additional file 3 — Table S1 - Primers designed for mapping transcripts to the ASGR-carrier chromosome. Microsoft word file: ASGR-Carrier Chromosome transcript primers.doc contains a table with primer sequences used for experiments to map ovule transcripts to the ASGR-carrier chromosome and the ASGR locus with annealing temperatures. [file 1471-2164-12-206-S3.DOC]

Table S1. ASGR-Carrier Chromosome transcript primers.

| **Oligo Name** | **Primer #** | **Sequence (5'-3')** | **Tm (°C)** |
| --- | --- | --- | --- |
| Ps26_c10331_F | 1476 | ACTAGGGGAAAGAAAGTCATCCTC | 60 |
| Ps26_c10331_R | 1477 | AAGCGTGTATCACTTCACGAAC |  |
| Ps26_c11544_F | 1478 | TACTTTTGGTGTGGTTTCGACC | 56 |
| Ps26_c11544_R | 1479 | CATACATGGACTACTGATGGCT |  |
| Ps26_c13157_F | 1480 | GGCTAGAACCGTCGAGCAT | 60 |
| Ps26_c13157_R | 1481 | TTCAGAGAATTAGGCCGAGGAG |  |
| Ps26_c13655_F | 1482 | GCTGCTGCTTCCTAGTTTCATT | 60 |
| Ps26_c13655_R | 1483 | CTAATTTTCAGGGCCAGGACAG |  |
| Ps26_c1372_F | 1484 | GCCATTGCGCTTCTGTAATAGT | 60 |
| Ps26_c1372_R | 1485 | AGCATACATGGTACATTGATCGAG |  |
| Ps26_c13922_F | 1486 | AGATCATTAGCCACTAGGATCGTC | 60 |
| Ps26_c13922_R | 1487 | AGAACAAGAACCTCCTGCTACAA |  |
| Ps26_c20942_F | 1488 | GAAGCATCTGTTCCGTTCACTC | 60 |
| Ps26_c20942_R | 1489 | TCCTGTGAATCAACAAGTTCAGC |  |
| Ps26_c24301_F | 1490 | CGTCCTGCTGCTCTTCTT | 58 |
| Ps26_c24301_R | 1491 | TGCTATTTCGGCAATTAACAGA |  |
| Ps26_c2448_F | 1492 | TATCGTGCCTGCTGGTGATAG | 60 |
| Ps26_c2448_R | 1493 | CTGTACTCCTTGATACGCAAGC |  |
| Ps26_c25664_F | 1494 | CAAAGTTGAGGACCCTGACAT | 58 |
| Ps26_c25664_R | 1495 | CATGCTTCGTTTTGGTCCATC |  |
| Ps26_c30198_F | 1496 | CATCACTACCCTCTTGATCTTTGC | 60 |
| Ps26_c30198_R | 1497 | AAGGACTCTGCTGTGAATGCTA |  |
| Ps26_c30691_F | 1498 | AAATGGACTTAGGCTTGCGTTG | 60 |
| Ps26_c30691_R | 1499 | ACAATACATCAATACCCAGTTGCC |  |
| Ps26_c3546_F | 1500 | CCGCGACAACATATCAACACAT | 60 |
| Ps26_c3546_R | 1501 | CGGATGAGAAAACAACAGCCAT |  |
| Ps26_c3993_F | 1502 | CCGTGGAAAACTTACTGATGGG | 60 |
| Ps26_c3993_R | 1503 | AGCAACAAGTTCCGTGTCAAAT |  |
| Ps26_c4364_F | 1504 | CAGATCACCCAGATCACCAC | 60 |
| Ps26_c4364_R | 1505 | AAGCTAAATCCTGGTGGAAAGA |  |
| Ps26_c5080_F | 1506 | TTAGGGACTGATGACACAAGGG | 61 |
| Ps26_c5080_R | 1507 | GGCATCAACCAGGACAATACTC |  |
| Ps26_c5781_F | 1508 | TGTTGTAGTTCGGTTTCTGCTC | 60 |
| Ps26_c5781_R | 1509 | TCCCATTTCAACCTTTATGCCAG |  |
| Ps26_c583_F | 1510 | CTACGACGACGACTACTGATCC | 62 |
| Ps26_c583_R | 1511 | ATTTTGCAGTGCTCAACTCCTC |  |
| Ps26_c8165_F | 1512 | TTCTTCTTCTTCACCACCAAGG | 60 |
| Ps26_c8165_R | 1513 | GCTCTTGCTGTTAGGTGTTGTT |  |
| Ps26_c9369_F | 1514 | GGCATACCAGCAAACCAAGTTA | 61 |
| Ps26_c9369_R | 1515 | ATCATCACATGCTTCAAACAGC |  |
| Ps26_c2339_F | 1528 | TAAAAAAGGGGAGGATGA | 55 |
| Ps26_c2339_R | 1529 | AGAAAAGCAGGATTTATAGTGT |  |
| Ps26_c1279_F | 1530 | TTTAAGTACCTTCTGTTTGGAG | 55 |
| Ps26_c1279_R | 1531 | TGGCTTTTACCTTGGTCT |  |
| Ps26_c7587_F | 1532 | TGATGAATTATTGGTGGTCA | 55 |
| Ps26_c7587_R | 1533 | GTTGTTTATTCATGTCCTTAATT |  |
| Ps26_c2785_F | 1534 | ATGAGATATATATGTAACC | 50 |
| Ps26_c2785_R | 1535 | GTAATAATCTTCGTGTTG |  |
| Ps26_c17388_F | 1538 | TCATCATCTTCGTCCTGG | 55 |
| Ps26_c17388_R | 1539 | AGAAGATCGACACACACAC |  |
| Ps26_c3455_F | 1540 | GGATGTGCTGTTGCCCTG | 55 |
| Ps26_c3455_R | 1541 | CAAGCAGCAGTTAACAGACTT |  |
| Ps26_c1312_F1 | 1542 | CTGTCGCTGTTCAACTAC | 55 |
| Ps26_c1312_R1 | 1543 | GAGCTAGACAACACATCA |  |
| Ps26_c6192_F | 1546 | GCGCCTTGTGCCAAAGTC | 60 |
| Ps26_c6192_R | 1547 | TTCTCACTCCCTCCAGCC |  |
| Ps26_c338_F1 | 1548 | GCTTTGGCATTGTTTGTG | 55 |
| Ps26_c338_R1 | 1549 | AGGTATTGCAATGCAAAGA |  |
| Ps26_c33813_F | 1565 | AATAAACTAGTACTGCAATCTGC | 55 |
| Ps26_c33813_R | 1566 | GTACGGTGTTCAAATAATGTG |  |
| Ps26_c1422_F | 1567 | GTTAGATTGCTGTTTTGATG | 55 |
| Ps26_c1422_R | 1568 | ATGAGCGCAATTTGAACA |  |
| Ps26_c6131_F2 | 1571 | CTCGGCAAAGCAACAAGCGG | 61 |
| Ps26_c6131_R2 | 1572 | CGTATTGGGAAACAACACGC |  |
| Ps26_c1472_F | 1573 | TCCATAAAACTAAAGGCTATGT | 54 |
| Ps26_c1472_R | 1574 | TTTGATGGATTACTATGTAAACC |  |
| Ps26_c2388_F | 1575 | TCGAGCTGTTAAAGGCGTGTCA | 61 |
| Ps26_c2388_R | 1576 | CCTTTGATAGTACATCAGGCCG |  |
| Ps26_c2405_F | 1577 | CATCTGTCGTCTCCGTGTC | 61 |
| Ps26_c2405_R | 1578 | CTCTTGCTGATGAGTATGGG |  |
| Ps26_c15085_F | 1579 | CATTATTTTAGAATTCTTGACTCC | 54 |
| Ps26_c15085_R | 1580 | CAAATATCATTCACAGTTCAACG |  |
| Ps26_c2009_F | 1581 | AACGCCAATTGATCACACGAGC | 61 |
| Ps26_c2009_R | 1582 | TCAGGCTGCAGCTGCTGATG |  |
| Ps26_c1406_F | 1583 | CCGATGGGGGTGATTTAATG | 61 |
| Ps26_c1406_R | 1584 | CGGTGACCATCTAGTAACAGGA |  |
| Ps26_c194_F2 | 1604 | AAACATCATATAGATCCGG | 53 |
| Ps26_c194_R2 | 1605 | ATTTTCCCTAATTCTCAGAG |  |
| Ps26_c1580_F | 1628 | TGGAGTCATTGTGGGCATCA | 60 |
| Ps26_c1580_R | 1629 | CATGGAGTCTCGGCAGTAGG |  |
| Ps26_c10535_F | 1630 | CACTTGTTGGTTCTTTCTGTTGG | 57 |
| Ps26_c10535_R | 1631 | TTAAGTGGTTCGTCGGCATA |  |
| Ps26_c18163_F | 1632 | ACAGAGGCAAGCTCCATAGC | 59 |
| Ps26_c18163_R | 1633 | AGGGTGCTCGACTATTGGTT |  |
| Ps26_c1878_F | 1634 | ACCATTCCGTCATCTCTCGG | 59 |
| Ps26_c1878_R | 1635 | ATTATCGGCGTGTAAGGCCA |  |
| Ps26_c19109_F | 1636 | GGCTTCAGTTTAGCGTTTGCT | 60 |
| Ps26_c19109_R | 1637 | AAGAATCATGCCAACACGCC |  |
| BC8_c547_F | 1638 | CACAAAGCTAAGAAGCGGTGAA | 60 |
| BC8_c547_R | 1639 | CACAAGGCCCACCAATGTTTAT |  |
| Ps26_c2807_F | 1640 | ATTACCGGCGTGTCACTTATCT | 60 |
| Ps26_c2807_R | 1641 | CGCATGGAAGCAGAAGGTTTAT |  |
| Ps26_c2838_F | 1642 | TTACCGGACATTGTGAGGAACG | 58 |
| Ps26_c2838_R | 1643 | CCAGAGTATTTGGTTCCCATCT |  |
| Ps26_c28392_F | 1644 | CTGTAGTACGGCACGAGTGG | 60 |
| Ps26_c28392_R | 1645 | CAAGTTCTCCGACTGCTTGAAA |  |
| Ps26_c3609_F | 1646 | GCAGACAGTTGGATGACATTGG | 58 |
| Ps26_c3609_R | 1647 | ACTAAAGGAGAGGCGCTGTAAA |  |
| Ps26_c3656_F | 1648 | TCGGAAACCAGCAGTCAATCTA | 59 |
| Ps26_c3656_R | 1649 | CATGTGTTGTATTTGGCGTTCC |  |
| Ps26_c4150_F | 1650 | GAGTCAGTCAAGAAGTGGAGGA | 60 |
| Ps26_c4150_R | 1651 | TTTAGCCCTTTACGACCACCAT |  |
| Ps26_c5210_F | 1652 | GGGTTTCAGGTTTGATTTCGGT | 60 |
| Ps26_c5210_R | 1653 | GCAAGACCGTCATACTTCATTCC |  |
| Ps26_c5851_F | 1654 | CAGAACAGCCACTTTGCTCTAA | 59 |
| Ps26_c5851_R | 1655 | GCTCCATTGGGACATACACATC |  |
| Ps26_c6373_F | 1656 | CTGACACCATAAACAGCAACCC | 60 |
| Ps26_c6373_R | 1657 | CTTCTACAACGACAAGAACCCG |  |
| Ps26_c6744_F | 1658 | TGCAACCTGTTAGGTGTCTTCT | 60 |
| Ps26_c6744_R | 1659 | CCAGACGGTGTACTAATCACGA |  |
| Ps26_c704_F | 1660 | CGTGATGACAAAGGACGAGTTC | 58 |
| Ps26_c704_R | 1661 | GGACGCCTTCCTTCTTCTTGTA |  |
| Ps26_c8378_F | 1662 | ACAGCAAATGAGCATCCAAACA | 60 |
| Ps26_c8378_R | 1663 | TAGGCATTGGAGATGGTGTCAA |  |
| Ps26_c9776_F | 1664 | TGTGATATGTGTGCCTAGCTGT | 60 |
| Ps26_c9776_R | 1665 | AATACGGCAAATGATGCCAAGT |  |
| Ps26_c14318_F | 1666 | TTGGAGTGCAATGGCTTCAAAT | 60 |
| Ps26_c14318_R | 1667 | ACTGACTTTGACACCCTCTGTT |  |
| Ps26_c21597_F | 1668 | TTGTGCTCATACGTTGGTGACG | 60 |
| Ps26_c21597_R | 1669 | AACGGCAAGATTCAGAAACACC |  |
| Ps26_c2552_F | 1670 | GAAGAGGATTGCCAAGAAGCAG | 58 |
| Ps26_c2552_R | 1671 | TCGACTGATCCAACACAGAACA |  |
| Ps26_c1406_f1 | 1680 | TCAGTTAGGCAGTGACCTGT | 59 |
| Ps26_c1406_r1 | 1681 | TGAAAGTCGAACTATAGAAGTCCCA |  |
| Ps26_c1472_f1 | 1682 | AGTCGGACAGCTTCTTGGAA | 60 |
| Ps26_c1472_r1 | 1683 | ATTTCTCTCCCGAACACCCC |  |
| Ps26_c150585_f1 | 1684 | CAGTCCATGCAACAACCTGG | 60 |
| Ps26_c150585_r1 | 1685 | GCTGTCGGAGCAAACTCAAA |  |
| Ps26_c1580_f1 | 1686 | TTCCCCATTCATCTCGCTGT | 60 |
| Ps26_c1580_r1 | 1687 | AACCAGCACACATGGAGTCT |  |
| Ps26_c18163_f1 | 1688 | TGATCTAGGTCTTTGCAAGTATTAGT | 55 |
| Ps26_c18163_r1 | 1689 | CCTAACAGAGGCAAGCTCCA |  |
| Ps26_c1878_f1 | 1690 | TAGCCAGCAAAAGGTAATCTCC | 55 |
| Ps26_c1878_r1 | 1691 | TTAGCGAGATTATCGGCGTG |  |
| Ps26_c19109_f1 | 1692 | TGCTGCTTTTTGTCAGAGTTCG | 55 |
| Ps26_c19109_r1 | 1693 | GTGCTTTCACCATTATCGTCCA |  |
| Ps26_c20942_f1 | 1694 | GGCTCAGAAAAAGAAGCATCTG | 55 |
| Ps26_c20942_r1 | 1695 | CACGGGTTTCAAATTGAAATTG |  |
| Ps26_c22381_f1 | 1696 | GTGAGGCAGGCCATATTTTGT | 55 |
| Ps26_c22381_r1 | 1697 | AAATCTTCGTCGAGACGTCGA |  |
| Ps26_c2405_f1 | 1698 | AAGACCAGTGATTCCACCAA | 55 |
| Ps26_c2405_r1 | 1699 | CAGCAAGATTTGGTGTTACTGC |  |
| Ps26_c24301_f1 | 1700 | TCAGTTAATGTAAACTTTGTTGCACTG | 55 |
| Ps26_c24301_r1 | 1701 | TGAAAAATGCTATTTCGGCA |  |
| Ps26_c25664_f1 | 1702 | AGAGAGGATACGGCTAACCA | 55 |
| Ps26_c25664_r1 | 1703 | TCATCTTCAACAACCACATGA |  |
| Ps26_c28392_f1 | 1704 | TCGTCCTGCTCCTGCCTCAA | 55 |
| Ps26_c28392_r1 | 1705 | CGGATCTCATCATGCCCATA |  |
| Ps26_c30198_f1 | 1706 | ACCCTCTTGATCTTTGCAACAG | 55 |
| Ps26_c30198_r1 | 1707 | TGGTGCTAAGCTTATGATCCCT |  |
| Ps26_c704_f1 | 1708 | TCCCCGATCAGGTAGCAAAC | 60 |
| Ps26_c704_r1 | 1709 | GCCGCTCTTGTTTGTCAGG |  |
| Ps26_c3656_f1 | 1710 | CTGCCACTTTGATGTATTGACG | 55 |
| Ps26_c3656_r1 | 1711 | CAATAATGCCCTGGCATGTC |  |
| Ps26_c3993_f1 | 1712 | GCGTAAACCATTTACTCAGAGC | 59 |
| Ps26_c3993_r1 | 1713 | GATGAGTTTGCTCCAGGACG |  |
| Ps26_c4150_f1 | 1714 | TCAAGAACTTGTGCTCAGATCC | 59 |
| Ps26_c4150_r1 | 1715 | GCTTCTGGTAACAACACACCA |  |
| Ps26_c4364_f1 | 1716 | ACAACGGTGTCACCAGATCA | 60 |
| Ps26_c4364_r1 | 1717 | CTCAGTGCTGTCGGTGTAGT |  |
| Ps26_c5781_f1 | 1718 | AGGTCTTTGTCAGGTTTTAGATTGA | 60 |
| Ps26_c5781_r1 | 1719 | TGGCAGATCAGAATACCTCCC |  |
| Ps26_c6192_f1 | 1720 | TTTGTTGCCTTTGTAAGCGC | 55 |
| Ps26_c6192_r1 | 1721 | ACTCCCTCCAGCCAAGTAGAAC |  |
| Ps26_33813_f2 | 1724 | GTGCGAAGCAAATAAGTGCCT | 60 |
| Ps26_33813_r2 | 1725 | CATGCTGGCATGAAGTTGTTCA |  |
| Ps26_c5080_f2 | 1744 | AAGGTGTGCTTTGGTGTTCTCC | 60 |
| Ps26_c5080_r2 | 1745 | CGATCCTTCCTTCTCTAAAGAGCG |  |
